# A qualitative study on the impact of caring for an ambulatory individual with nonsense mutation Duchenne muscular dystrophy

**DOI:** 10.1186/s41687-021-00344-8

**Published:** 2021-08-10

**Authors:** Kate Williams, Ian Davidson, Mark Rance, Katharina Buesch, Sarah Acaster

**Affiliations:** 1 Acaster Lloyd Consulting Ltd, 16 Upper Woburn Place, London, WC1H 0BS UK; 2 PTC Therapeutics Ltd, Building 2, Ground Floor, Guildford Business Park, Guildford, UK; 3 PTC Therapeutics Switzerland GmbH, Tower 2, Turmstrasse 28, CH-6312 Steinhausen/Zug, Switzerland

**Keywords:** Duchenne muscular dystrophy, Nonsense mutation, Caregiver, Carer, Qualitative, Ataluren

## Abstract

**Background:**

Duchenne muscular dystrophy is a rare genetic neuromuscular disorder, which can result in early death due to disease progression. Ataluren is indicated for the treatment of nonsense mutation Duchenne muscular dystrophy, in ambulatory individuals aged two years and older. This study explored the impact of caring for an ambulatory individual with nonsense mutation Duchenne muscular dystrophy, as well as the impact of treatment with ataluren on the caregiver experience, using retrospective recall.

**Methods:**

Qualitative interviews were conducted with caregivers in the UK. Interviews were conducted by telephone, were recorded and transcribed. Data were analysed using thematic analysis and saturation was recorded.

**Results:**

Ten interviews were conducted with parents of individuals aged 4–19 years. Caregivers reported proximal impacts (physical, emotional, time-related), and distal impacts (work, relationships, social life) of caring for their sons. The relationships between these impacts were illustrated in a conceptual model. Changes to the caregiver experience since initiation with their son’s treatment were discussed.

**Conclusion:**

Caring for an ambulatory individual with nonsense mutation Duchenne muscular dystrophy has a substantial multifaceted impact on caregivers. Treatments which have the potential to improve symptoms or delay progression, may also have a positive impact on the quality of life of caregivers.

**Supplementary Information:**

The online version contains supplementary material available at 10.1186/s41687-021-00344-8.

## Introduction

Duchenne muscular dystrophy (DMD) is an X-linked neuromuscular disorder characterised by progressive muscle degeneration [1]. It leads to delayed motor milestones, typically progressing to loss of ambulation at approximately 12 years of age, followed by severe cardiac and respiratory complications [1, 2]. The pooled global prevalence of DMD is around 7.1 cases per 100,000 males [3]. In approximately 10–15% of cases, DMD is caused by a nonsense mutation on the DMD gene (nmDMD) [4]. While there has been substantial progress in DMD clinical research in recent years, ataluren is the only licensed treatment for individuals with nmDMD. Ataluren 40 mg/kg/day is indicated for the treatment of nmDMD in ambulatory individuals aged ≥2 years in member states of the European Union [5].

DMD is a lifelong condition that begins in infancy and most individuals live at home with their parents throughout the course of their lives [6]. As a result, they receive the majority of their day-to-day care from informal unpaid family caregivers [6]. Depending on the extent of the individual’s limitations, caregivers may be required to provide support for basic activities of daily living (e.g. washing and dressing), instrumental activities of daily living (e.g. managing appointments), as well as emotional support. Determining the extent of the tasks undertaken by caregivers of individuals with DMD, and the impact this has on their health-related quality of life (HRQoL), is crucial to understanding the potential burden and unmet need in this population.

A systematic review of caregiver burden in DMD identified a substantial impact of informal caregiving, including impaired health and HRQoL, poor sleep, reduced family function, increased risk of depression, elevated levels of stress, sexual dysfunction, as well as an impact on work life and productivity [6]. The majority of included studies used survey methodology and evaluated caregiver burden using standardised measures of caregiver burden or generic HRQoL. A more recent systematic review examined health state utilities associated with caring for individuals with DMD reported in the literature, all of which were derived using the EQ-5D [7]. This found that caregiver utilities ranged from 0.87 in a study of 80 caregivers of adults with DMD (96% on ventilatory support) [8], to 0.71 for caregivers of children in a study of 154 caregivers of children with DMD [9]. However, in general, utility values were lower for caregivers of individuals who were non-ambulatory, suggesting that the value of 0.87 may reflect adaptation or coping. Among caregivers of individuals who were ambulatory, utility values ranged from 0.85 for caregivers of individuals who were early ambulatory and 0.83 for caregivers of individuals who were late ambulatory [10, 11]. Even with this quantitative data, there is no evidence that the EQ-5D can adequately capture the full burden of caring for individuals with DMD, and this review highlighted the need for further examination of caregiver burden using different methodologies [7].

Qualitative research provides in-depth information on the experiencing of caring which may not be captured by questionnaires. This is particularly valuable in rare diseases, where very little is known about the patient experience of living with a condition. Very few qualitative studies have explored the impact of caring for an individual with DMD in the caregiver’s own words, and none have specifically examined the impact of caring for an individual who is still ambulatory. Although seven studies identified in the systematic review of caregiver burden included interviews [6], only one used qualitative methods and this was focused on the impact of being a sibling to an individual with DMD [12]. A more recent qualitative study, not included in the systematic review, used serial qualitative interviews to examine the impact of caring for adult sons with DMD [13]. This reported strengths and weaknesses associated with caring for the individual with DMD; strengths included family support and confidence in parenting ability, and weaknesses included the anticipation of ageing with the ongoing burden of caring, regrets, sharing responsibilities versus having a fixed role as a primary caregiver, and economic burden.

To-date, no qualitative studies have examined the impact of caring for an ambulatory individual with nmDMD, and none have explored the potential impact of treatments on caregiver burden. This study explored the impacts and challenges experienced by caregivers of ambulatory individuals with nmDMD, as well as the impact of their son’s treatment with ataluren.

## Materials and methods

### Design and participants

This was a cross-sectional qualitative interview study with caregivers of ambulatory individuals with nmDMD treated with ataluren in the United Kingdom. The aim of the interviews was to explore the impact of caring for an ambulatory individual with nmDMD before they started treatment with ataluren, using retrospective recall. Another aim was to explore how treatment with ataluren had impacted their experience of caring.

### Study materials

A semi-structured interview guide (Supplementary file 1) was developed based on the published literature on the HRQoL of individuals with DMD and through consultation with clinical experts in the UK and Germany and two patient advocacy groups (PAGs) in the UK (Muscular Dystrophy UK and Action Duchenne). The clinical experts included two medically/surgically trained clinicians, with a combined clinical experience of more than 10 years, and medical affairs/clinical development experience of more than 15 years, with a focus on muscular dystrophies. The PAG input included feedback from a caregiver of an individual with DMD. The interview guide comprised mainly of open-ended questions on the impact of caring for an ambulatory individual with nmDMD (impacts on the individual with nmDMD were also explored, but are not included in this paper).

A background questionnaire was developed to collect socio-demographic information, as well as information on the time spent on different types of care and the support they receive (Supplementary file 2). This also included questions which allowed the individuals with nmDMD to be characterised into one of three ambulatory health states according to the natural history model developed by the University of Leicester (early ambulatory: can rise from supine, stand and walk 10 m; late ambulatory: can stand and walk 10 m; transfers: can stand) [14]. Throughout this paper we use the term ambulatory to refer to these three health states.

### Ethics review and approval

This study was reviewed and approved by the WIRB-Copernicus Group Independent Review Board (IRB tracking number: #20193514).

### Recruitment and interviews

Participants were recruited by PAGs using purposive sampling. The study was advertised via newsletters and social media and interested individuals were encouraged to get in touch using the contact details provided. Participants who expressed an interest in taking part were asked a few brief screening questions by email to check they met the inclusion criteria of (i) being the main caregiver (at least 50% of caring) of an individual with nmDMD treated with ataluren in the UK, (ii) aged 18 years or over, (iii) live in the UK, (iv) willing and able to provide informed consent. Participants were sent an information sheet about the study, along with the background questionnaire to complete and return by email. Due to the limited pool of potentially relevant participants, all of those who were eligible were included and no additional sampling criteria were used.

Interviews were conducted by three interviewers (KW, NP and KG), all with postgraduate degrees in psychology (two master’s and one PhD) and more than 25 years combined qualitative research experience. None of the participants were known to the interviewers. All interviews were conducted by telephone between 10 February 2020 and 20 March 2020 (prior to the UK COVID-19 lockdown). Verbal informed consent was taken at the start of the interview and was recorded. Interviews followed the semi-structured interview guide and lasted around 90 min. Participants were informed that they could refuse to answer any questions they did not wish to answer and were given the opportunity to speak freely and honestly. If participants answered inconsistently, they were probed to clarify any ambiguities.

The interview recordings were transcribed, and the transcripts were de-identified using participant identification numbers and names were removed prior to analysis. These were stored on a secure server, separate from any participant names and contact details.

### Analyses

Data from the background questionnaire were summarised using descriptive statistics. Data from the interviews were analysed using thematic analysis in MAXQDA. As with the interviews, all researchers involved in the analysis had postgraduate degrees in psychology (two master’s and one PhD), with more than 30 years combined qualitative research experience. Two researchers (KW and NP), who also conducted the interviews, read all the transcripts and developed a coding framework based on the topics covered in the interview guide. They then independently coded the same transcript (C101) and discussed discrepancies. A third researcher reviewed and provided input (SA). The coding framework was revised following this discussion and the remaining transcripts were coded by KW (*N* = 1) and NP (*N* = 8) using the updated framework. To enhance the trustworthiness of the research, after each transcript was coded, the two researchers discussed and additional amendments were made to the coding framework as needed. When codes were added or changed, the previously coded transcripts were reviewed, and the new codes were applied as needed. KW conducted a final quality check of al transcripts to ensure the completeness and accuracy of the coding.

The codes were then grouped into themes to describe the experience of caring for an ambulatory individual with nmDMD. A conceptual model was developed to illustrate the relationship between these themes (impacts). The caregiver impacts were described in boxes and arrows were used to indicate the direction of the relationships between the impacts. These relationships were based solely on the qualitative data.

Best practice in qualitative research is to keep conducting interviews until data saturation is reached. Data saturation has been defined as the point at which no new insights are obtained, or no new themes are identified in the data [15]. The number of participants needed to reach saturation is largely driven by the complexity of a concept and the diversity of the population who share the commonality of interest. A saturation matrix was used to monitor the frequency of reported concepts across the interviews, where the concepts were listed in rows and the columns were the interviews in order of completion [16].

## Results

### Sample characteristics

Ten caregivers took part in the interviews, all of whom were parents to an ambulatory individual with nmDMD. The characteristics of the caregivers are shown in Table 1.

**Table 1:** Caregiver characteristics (*N* = 10)

Characteristic	Mean (SD)	Range
**Age (years)**	**44 (5.4)**	**26–52**
**Relationship to individual with nmDMD**		**N (%)**
Father		5 (50)
Mother		5 (50)
**Ethnic background**		
White		10 (100)
**Education**		
O level/GSCE or equivalent		3 (30)
A Level or Highers		1 (10)
Higher education below degree level		0 (0)
University degree or higher		6 (60)
**Employment**		
Employed full-time		6 (60)
Employed part-time		2 (20)
Full-time homemaker/caregiver		2 (20)
**Others in household**		
Partner		9 (90)
Other children		8 (80)
**Changes to work in order to care for individual with nmDMD**^a^		
Reduce working hours		4 (40)
Change jobs		1 (10)
Stop working		1 (10)
None of the above		5 (50)
**Help with caring**		
Yes		7 (70)
No		3 (30)
**Time spent caring for individual with nmDMD**	**Mean hours/week**	
	**Practical care**	**Emotional care**
Participant	33	26.3
Other parent/partner (N = 7)	27	18
Other family member (N = 1)	12	2
Paid personal assistant (N = 1)	10	5
Other (N = 1 learning support assistant)	36	36

^a^Total is > 10 as participants could select multiple option. *SD* standard deviation; *nmDMD* nonsense mutation on the Duchenne Muscular Dystrophy gene

The data saturation matrix (Table 2) indicated that saturation was reached, with 70% (17/24) spontaneously reported in the first 50% of the interviews and 92% (22/24) in the first 70% of interviews. In addition, no more impacts were spontaneously reported in the final interview.

**Table 2:** Data saturation matrix

Caregiver impact	Participant number^*^									
	C102	C101	C103	C104	C105	C106	C108	C107	C109	C110
**Physical**										
Tiredness/exhaustion	P					**S**	S		S	
Lifting/carrying	**S**	S		S						
Back pain/aches	**S**	S			S					
Sleep	P	**S**			P					
**Emotional**										
Upset/sad		**S**	P		S	S	S	S		S
Feeling hopeless	**S**	S	S			S	S	S	S	S
Stress		**S**	S				S	P	S	S
Worry/anxiety	**S**	S			S		S	P	S	
Guilt		**S**			S					
Loneliness/isolation							**S**			S
Positive emotions	**S**				S		S	S		S
**Time-related**										
No time for self	P					**S**	S			
Appointments/admin		**S**			S		S		S	S
**Work**										
Time off work		**S**			S					P
Reducing hours/stopping hours	P				**S**		S	S		
Flexible/home working		P							**S**	
Conversations with colleagues		**S**								
**Relationships**										
With partner	**S**	P					S		S	P
Friendships		P			**S**			S	S	S
**Social**										
Cancelling plans	**S**	S								
Limited social life/hobbies		**S**			S		S			S
Difficulty finding childcare									**S**	S
Difficulty socialising with child						**S**	S	S		
Needing to plan ahead						**S**				

^*^Participant numbers are presented in the order the interviews were conducted. S = spontaneously reported impacts, *P* probed impacts. Impacts were considered spontaneously reported unless explicitly probed (e.g. If they were asked about any social impacts and they reported cancelling plans, then ‘cancelling plans’ was considered a spontaneously reported impact. Bold letters indicate the first spontaneous report

### Overview of caregiver impacts prior to their sons starting treatment with ataluren

**Fig. 1:**
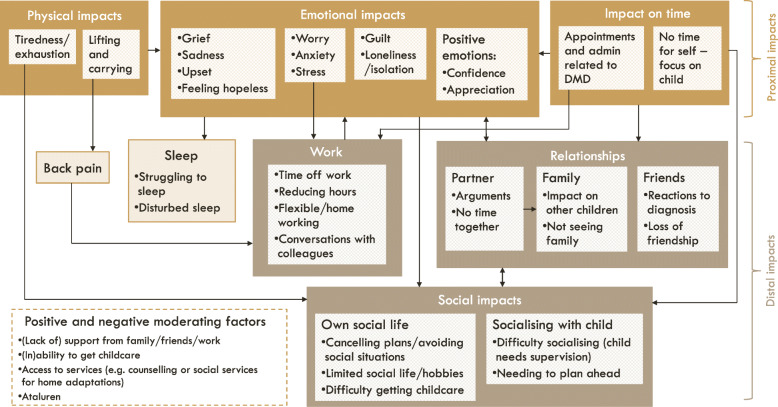
Conceptual model on the impact of caring for an individual with ambulatory nonsense mutation Duchenne muscular dystrophy Footnote: The conceptual model is designed to be read from the top, where there are the most proximal impacts, to the bottom, where there are more distal impacts. The arrows show the relationships between the concepts, which are either unidirectional (e.g. work had an impact on emotional wellbeing) or bidirectional (e.g. relationships impacted social impacts and vice versa). Some relationships are between the larger external boxes (e.g. emotional impacts had an impact on sleep), whereas others are between the internal boxes (e.g. partner relationships impacted family relationships)

The relationships between the impacts are shown in a conceptual model in Fig. 1. The caregiver impacts were categorised as proximal or distal based on how directly related they were to the experience of caring for an individual with nmDMD. This categorisation does not imply any difference in importance between the two groupings. Proximal impacts included physical, emotional and time-related impacts, which were each reported as being directly related to caring for the individual with nmDMD. For example, caring for an individual with DMD had a direct impact on the amount of time caregivers had for themselves. These proximal impacts had a subsequent impact on more distal impacts, including work, emotions, relationships, and social activities. These distal impacts were one or more steps removed, for example, caring for the individual with DMD did not have a direct impact on work, but it had an impact on emotional wellbeing, which had an impact on work. Each of these impacts is described in further detail below.

### Proximal impacts

#### Physical impact

Several caregivers talked about how they needed to lift and carry their sons and that this impacted them physically. For example, they described how they needed to constantly lift and carry their son throughout the day, including carrying them upstairs and lifting them in and out of the car or in and out of bed.

*“Physically it had an impact on me because I was carrying him a lot, so I was carrying him up the stairs, carrying him out of the school, carrying if we were out as a family carrying him out of the car, so doing lots of lifting”* – C101

Several participants described how the strain of constant lifting and carrying had resulted in them experiencing back problems, including sciatica, back ache and muscle spasms.

*“I’ve been off work a couple of times, with back aches” –* C105

One caregiver mentioned that they worried about injuring their back and not being able to lift and carry their son anymore.

Another widely reported physical impact was tiredness and exhaustion. Several caregivers described how they felt drained and exhausted from looking after their son all day.

*“It would take more energy to get him to do something so it would be draining and I’d feel very tired at the end of the day” –* C108

This included both physical exhaustion, for example, from lifting and carrying, and mental exhaustion from having to plan everything in advance. Some reported that caring impacted their sleep, for example, one participant reported that their son sometimes woke up at night with leg cramps and this resulted in them having disrupted sleep.

*“I mean you have a broken night’s sleep if he woke up with cramps in his legs” –* C102

However, it was acknowledged that most parents they knew had sleep disturbance.

#### Emotional impact

A wide range of emotional impacts were reported by caregivers in this study. Among the most common were grief and sadness/upset. Several caregivers talked about the grief they had experienced on learning about their son’s diagnosis, for example, one caregiver described how they were in shock following their son’s diagnosis and that it took them 6 months to get over the initial pain and begin to process what it meant. Some also talked about grieving the future they imagined for their son and coming to terms with their new life.

*“We went through what is known as the grieving process for someone who’s still alive” –* C105

Other caregivers described a general feeling of “enormous sadness” at their son’s diagnosis and at watching them deteriorate.

*“I would say the main feelings would have been sadness, enormous sadness” –* C107

On a day-to-day level, some caregivers talked about how they would become upset if their son was upset or when trying to discuss issues with their partner. Some caregivers described feelings of hopelessness because there was nothing they could do for their son.

*“Just feeling a little bit hopeless about everything and sort of thinking, “I don’t really know, there’s nothing I can do” so you just end up feeling a bit hopeless” –* C101

One caregiver reported that they had been diagnosed with depression and had been prescribed anti-depressants.

Other commonly reported emotions were anxiety, worry and stress, in relation to both immediate concerns and the future. For example, several caregivers talked about day-to-day worries about their sons overexerting themselves or becoming injured doing a certain activity or getting an infection and getting sick.

*“Just constantly worrying if I’m going to get a call he’s been rushed into hospital or something like that, just my mind overthinking, overrunning all the time, thinking that something’s going to happen, and always thinking the worst” –* C105

Others talked about a general worry and anxiety about the future in relation to their son’s condition declining and the lack of treatments available. Some caregivers also described feeling anxious and stressed about whether their sons would be able to get access to ataluren.

A smaller number of caregivers reported feelings of loneliness and isolation due to their son’s nmDMD.

*“It was quite isolating and lonely more than anything” –* C108

The social isolation generally related to avoiding certain social situations that were difficult because of their son’s nmDMD. On the other hand, the loneliness was generally related to feeling that others did not understand their situation and what they were going through. A couple of caregivers reported feelings of guilt, for example, one woman said she felt guilty for pushing her son too hard before he was diagnosed with nmDMD. Another caregiver described frustration at not knowing if their son would be able to get access to ataluren. Some caregivers also discussed positive emotions around being a caregiver to their sons. For example, several participants mentioned that it had resulted in more quality time spent together as a family.

*“My confidence beforehand, I would be the type of person who if I walked into a room I wouldn’t speak to anybody because I would be so shy but when it came to fighting for [the name of child], there was a new confidence there that I had never come across before, and I actually surprised myself!” –* C102

#### Time-related impact

Most caregivers described how caring for their son took a substantial amount of time and that this left little time for anything else. For example, one caregiver described the large number of hospital and physiotherapy appointments that their son needed to attend and how these took up a lot of time, especially with the travel back and forth.

*“I would say the biggest impact is hospital appointments and physio appointments. You have to fit your work around these because they all occurred during the working day and in the week, Monday to Friday. So, lots of time was spent going to hospitals or getting cover to go to the hospital appointments, that was the biggest impact” –* C110

Others reported having to spend time on administrative tasks relating to their son’s nmDMD, including making phone calls and managing appointments. Several caregivers reported that they had very little time for themselves as they were so focused on looking after their sons and taking them to their appointments.

*“You physically don’t have the time to do the things that you would normally do” –* C 106

### Distal impacts

#### Impact on work

Several caregivers reported that they had needed to make changes to their work including reducing their hours, changing jobs and stopping working altogether (Table 1).

*“I would work very minimum hours, I think at the time I was working in a school as a lunchtime supervisor so I’d maybe do two and a half hours a day just purely because I needed to be around for him, he was like a 24/7 type of job with him, giving him the support physically and mentally so it meant that I couldn’t have a career or a full-time job” –* C108

This was generally due to not having the time to work as well as look after their son and manage their appointments. For example, one caregiver said that they worked part-time from home in order to manage their son’s appointments and make phone calls during the day. Another said that they worked minimal hours as caring for their son was a full-time job. Others mentioned that they found it difficult to interact with colleagues following their son’s diagnosis with nmDMD, as they found it hard to relate to other people’s problems. Some caregivers reported that they had needed to take time off sick for physical and mental health issues that had arisen due to their son’s nmDMD. For example, one participant said they had 4 months off work due to anxiety and stress related to their son’s condition.

*“I ended up going off sick for about four months with anxiety and stress” –* C101

Another reported that they had needed to have time off work due to back ache as a result of lifting and carrying their son.

*“I’ve been off work a couple of times, with back aches. I have had three or four days off work”* – C105

#### Impact on relationships

Several caregivers reported that their son’s nmDMD had impacted their relationships with others, including their partner, their family, and their friends. Some caregivers commented that their son’s diagnosis had put a strain on their relationship with their partner because they were now so focused on their son they have little time to invest in their relationships. One caregiver reported that their partner had a different approach to managing their son’s nmDMD which had caused tensions in their relationship.

*“Myself and my husband had different ways of approaching things, so his would have been very much live in the here and now, where I was constantly looking towards the future and things that could go wrong and I think we just had different ways of approaching things…there was a few instances where we just, we didn’t agree on what the best plan forward was” –* C102

Another caregiver said that they had separated from their partner when their son was younger, so they had to manage their son’s condition on their own.

Some caregivers described how they had lost friendships as a consequence of their son’s nmDMD. While this was partly because their social lives were limited, some participants said that they had grown apart from friends because they felt their friends didn’t know how to discuss their son’s nmDMD or understand what they were going through. Similarly, one caregiver reported that they had grown apart from their brother because he was not very interested in their son’s nmDMD.

*“Even my own brother, we kind of drifted apart a little because he just wasn’t that interested in it and so we saw him very occasionally” –* C110

Some caregivers also talked about the impact of their son’s nmDMD on their other children, for example, one described how her daughter would get upset when they were upset and that it also affected her sleep.

On the positive side, some caregivers also discussed some of the ways in which their family and friends had been supportive, and others said they had made new friends with other parents of individuals with DMD.

#### Impact on social life

Most caregivers reported that their social lives were limited because of their son’s nmDMD. Some said that they cancelled plans or avoided social events because they weren’t feeling up to it mentally.

*“I wasn’t feeling great so I would cancel social events, I wouldn’t go to things” –* C101

One caregiver described how they became a “recluse” following their son’s diagnosis. Several caregivers reported that they didn’t have time for their own social life or hobbies as caring for their son took up most of their time.

*“I used to do a lot of triathlon but since [the name of individual with DMD]‘s diagnosis now, it’s six years now, that tapered off quite a lot. We spent a lot of time just as a family getting used to the diagnosis and obviously changed a lot of our habits. It’s changed what we do in terms of activities were a lot less social”* – C110

Others were unable to continue hobbies they had prior to their son’s diagnosis, as the hobbies were not suitable for their sons. For example, one caregiver said that they used to be a very sporty family, but this was no longer possible as their son was unable to join in with sports. Some caregivers reported that they found it very difficult to get childcare as their child’s needs were difficult for others to manage.

Some caregivers said that it was difficult to socialise with their son as he needed so much attention. For example, one participant commented that if they met up in the park with their friends, all the other children would play together so the parents could chat, but their son was unable to join in without support.

*“If we met friends and, say, went to a park, because he would still go to a playground then, most of the mums at that age could sit down and their children would just go off and play. Well, I couldn’t, I would always have to be with [the name of child] because he’d need a hand on any of the things he wanted to play on, he would always need help” –* C107

Others described how they felt it was sometimes easier to avoid social situations rather than having to discuss their son’s nmDMD or put their friends in difficult situations.

*“It was easier to stay away because we didn’t want to speak about it or put people in awkward positions…a lot of activities kind of fell by the wayside” –* C110

### Moderating factors

The extent of each of the impacts was influenced by a range of positive and negative moderating factors. For example, those caregivers with support from their family, friends, or work found it easier to manage their son’s nmDMD than those who did not have such support. Similarly, access to childcare had an impact on how able caregivers were to have time to themselves to socialise with their partner and friends. Access to support services was also an important moderating factor. One caregiver reported receiving counselling which had helped them deal with the emotional impact of caring for their son. Another reported receiving physiotherapy to help them manage their back pain caused with lifting and carrying their son. One caregivers commented that they may need support from social services in the future to help lift and carry their son as he gets bigger.

### Changes to caregiver impacts since their sons started taking ataluren

Several participants reported positive changes in their son’s condition following initiation with ataluren (not included in this paper), and these positive changes had a direct impact on the caregiver experience. Two caregivers reported that they did not need to lift or carry their sons as much as they no longer required the same level of physical support. One of these caregivers said this meant that they were not having so many issues with their back.

*“I mean physically it’s had an impact because I’m not carrying him as much so I’m not having to give him quite so much physical support, so my health is better in that sense because I’m, my back isn’t giving out so much” –* C101

Three caregivers said that they were less anxious and worried than before, because they had noticed ataluren was having a positive impact on their sons.

*“Once he started taking it I think it’s just made us all, I think I just feel sort of less anxious obviously, I can see that it’s having a positive effect on him, so it’s reduced my worry levels” –* C101

*“With [son]‘s behaviour improving and his ability to control himself improving, it meant that the daily stress and worry of how [son] interacts with other children and his brother was lifted significantly”* – C110

One caregiver said that they were now able to go to work without worrying about their son and were better able to focus on their own tasks.

*“I go to work now, and I don’t worry about what's happening at nursery, is he going to fall over? Am I going to get a phone call from the ambulance saying he’s in hospital? I’ll go to work and it will be, “Oh how’s [son] today at nursery? Has he done this, has he done that?” I’m not worrying, I’m able to focus more on my day to day. So I don’t feel like I’m worrying about him, because I know how well he’s doing” -* C105

Similarly, another participant reported that they were able to have more of a social life now because their son could now be left alone for several hours.

*“I’m able to have more of a social life, I can do more things. He can be left alone for you know hours and hours, I can go out for instance from say 9am until 5pm and [son] will cope perfectly fine at home without me or anyone here, so that’s a big change. So, yeah, I can do a lot more, going to work full time and just doing more or less normal day-to-day stuff that most other people would do now.” –* C108

Two caregivers described an overall positive impact of ataluren on their quality of life because they could see their son improve.

*“I think the worry is going to be there no matter what, it’s always going to be there because it’s a progressive disease. But even though it’s a progressive disease, with [ataluren] it’s like having a new lease of life, it’s like he’s been given an extra chance, he’s been given a few more years of walking, maybe longer. I’m aware of children on [ataluren] who are 11 or 12 and still walking and showing no signs of getting ready for a wheelchair yet. So I believe with his determination, and the way he carries on, that he’ll be one of these children that are still walking about. That’s what gets me through each day now, just watching him become this stronger, more determined than he was before, young boy” –* C105

One caregiver talked about a decline in their mental wellbeing which had occurred since their son had started taking ataluren. This was attributed to a decline in their son’s abilities rather than an impact of ataluren.

*“We [partner] both found 2018-19 ish quite a hard year emotionally with some of his decline in his abilities. I have seen a psychologist as well, the same one that [son] sees. My eldest son saw her because he was struggling as well, so it’s not only the impact it’s having on [son], it’s the impact it’s having on my older son as well. And then obviously that has an impact mentally on me. So, I don’t mind saying to you that when I was seeing the psychologist, she said to me that I was moderately depressed and I have started taking medication for that”* – C107

## Discussion

This is the first qualitative study to explore the impact of caring for an ambulatory individual with nmDMD and to illustrate the relationship between these impacts in a conceptual model. Proximal impacts included physical, emotional, and time-related impacts. These were linked to back pain, sleep, work, relationship and social impacts. These qualitative findings highlight the substantial burden of being a caregiver for an individual with nmDMD, even while they are still ambulatory. This may provide valuable insight to clinicians and regulators and help the DMD community illustrate the breadth of impact of the disease, and the unmet needs of caregivers. These findings also provide some qualitative evidence to support the disutility associated with caring for an individual with DMD found in previous studies [7, 10, 11].

Participants in this study were primary caregivers of individuals with nmDMD and reported doing at least 50% of the care. However, results from the background questionnaire indicated that the majority of participants (*N* = 7) also had support from a partner, other family member and/or paid personal assistant. Each of these were also reported to spend a substantial amount of time caring for the individual with nmDMD. This suggests that the impacts reported in this study are on the whole reflective of the experience of a caregiver with additional help. Although it was not explored specifically in this study, the burden on caregivers without help is likely to be even greater. These findings also indicate that multiple people may be required to provide sufficient care for ambulatory individuals with nmDMD. This should be considered when quantifying the extent of caregiver burden in this population. Another point to note is that previous studies have used the EQ-5D to evaluate caregiver burden [7–11], but this study highlighted a wide range of impacts that are not necessarily captured by this measure. Future research would benefit from exploring the ability of the EQ-5D to adequately capture caregiver burden in DMD and other caregiver populations.

This study also provided novel insights on the potential impact of treatment with ataluren on caregivers. Caregivers reported improvements in a range of areas following their son’s initiation with ataluren, including physical, emotional, work, and social impacts. Although it is not possible to know for certain whether these improvements are linked to their son’s taking ataluren, these findings highlight the potential impact of a treatment to extend beyond the individuals with nmDMD to those who care for them. One caregiver reported that their emotional wellbeing had worsened in the time since their son started taking ataluren, but this was attributed to the progression of their son’s nmDMD. As caregiver data is not routinely captured in clinical trials, these qualitative results may provide novel insights into the impact on the caregiver and support the shared decision making of families and their healthcare teams.

The conceptual model was directly based on the important concepts reported by caregivers in this study. Although some concepts in the model have been previously reported in the literature [6, 13], new concepts were identified in this study, including tiredness and fatigue, and the time burden associated with appointments and administrative tasks. This is also the first study to explore these impacts in depth and in the caregiver’s own words. As well as highlighting important impacts, the conceptual model illustrates the relationships between these impacts, demonstrating how burden in one area of life may impact other areas of life. Similarly, this can provide a useful tool for highlighting the potential impact of a treatment, as an improvement in one area may lead to an improvement in other areas.

While this study provides novel insights, it also had some limitations that need to be acknowledged. All participants in this study were caregivers of individuals with nmDMD who were currently taking ataluren. While this was necessary to evaluate the potential impact of ataluren on caregivers, it is possible that these individuals have different characteristics to those not eligible for ataluren. Although participants were also asked about their experience of caring for their sons prior to them taking ataluren, this relied on retrospective recall, so is not as reliable as including caregivers of individuals not on treatment. Our data saturation matrix indicated that data saturation was reached, but it is still possible that the findings may vary in different populations, for example, in non-parent caregivers. All interviews were conducted by telephone to avoid the need for caregivers to travel and make childcare arrangements. While this enabled us to speak to a larger sample of caregivers, non-verbal cues can be missed with telephone interviews and it can be harder to build rapport with participants. Nonetheless, all participants were engaged with the interviews and provided detailed responses. While a number of steps were taken to ensure the trustworthiness of the findings, it is possible that the experience of the individuals who responded to the study advert may differ from those who did not express an interest.

Despite these limitations, this is the first qualitative study to provide an in-depth exploration of the impact of caring for an ambulatory individual with nmDMD. As the impacts of caring likely change as an individual becomes non-ambulatory, and treatment benefits may expand or differ, future research could focus on extending this qualitative study to include caregivers and/or individuals with nmDMD who are non-ambulatory.

## Conclusions

Caring for an ambulatory individual with nmDMD has a substantial impact on the lives of caregivers. Treatments which improve symptoms and function in individuals with nmDMD have the potential to also improve the physical and mental wellbeing of caregivers, as well as their overall HRQoL. These data illustrate the breadth of impact of nmDMD, which may be valuable for clinicians and regulators, and informative for families in their shared decision making with healthcare teams. Extending the current study to explore the impacts of nmDMD, and potential benefits of treatment, across the full disease trajectory is a critical next step.

## Supplementary Information

**Additional file 1: Supplementary file 1.** Interview guide.

**Additional file 2: Supplementary file 2.** Background questionnaire.

## Data Availability

Available from corresponding author upon request.

## Abbreviations

DMD: Duchenne muscular dystrophy
nmDMD: Nonsense mutation Duchenne muscular dystrophy
HRQoL: Health-related quality of life
PAG: Patient advocacy group
SD: Standard deviation
