# Supplementary material for: A qualitative study on the impact of caring for an ambulatory individual with nonsense mutation Duchenne muscular dystrophy
Source: J Patient Rep Outcomes. 2021 Aug 10;5:71. doi: 10.1186/s41687-021-00344-8 (PMC8353428; doi:10.1186/s41687-021-00344-8)
Supplement: Supplementary file 1 — Additional file 1: Supplementary file 1. Interview guide. [file 41687_2021_344_MOESM1_ESM.docx]

Interview guide: Caregiver of an individual with nonsense mutation Duchenne muscular dystrophy

| INSTRUCTIONS TO INTERVIEWER (NOT TO BE READ TO THE PARTICIPANT)   - This interview guide is to be used for interviews with caregivers of individuals with nmDMD – please refer to the separate guide for patient interviews - The guide is intended to be used as the basis of discussion, but where possible the interview should feel like a conversation - Not all questions and probes need to be asked - throughout interview, only ask questions/use probes where appropriate, taking into account what has already been discussed, as well as the profile and age of the individual with Duchenne, and the serious nature of the condition for those affected - Use the field notes template to make notes so that you can return to topics later in the interview and avoid repetition - Instructions to the interviewer are in boxes like this one – do not read this text to the participant - Throughout this interview guide, we refer to the individual with Duchenne as a male and as the participant’s son. Please adapt this for caregivers of girls/women and those who are not parents. |
| --- |

# INTRODUCTION

Good (morning/afternoon/evening), my name is [Name] and I’m a researcher from Acaster Lloyd Consulting. We’ve been commissioned by PTC Therapeutics International Ltd to conduct this study looking at the impact of nonsense mutation Duchenne muscular dystrophy and treatment with ataluren (Translarna). If it’s ok with you, from now on I will refer to these as ‘Duchenne’ and ‘Translarna’.

Thank you for taking the time to participate in this interview. The purpose of this study is to understand how Duchenne impacts you and your son and how this may have changed when he started taking ataluren (Translarna). We are interested in understanding your perspective as a caregiver.

The interview will take up to 90 minutes. Everything you say will be kept confidential and only de-identified data will be shared or published beyond the study team. If you would prefer to keep the name of your son private, you can simply say ‘my son’ or use a pseudo name.

Some of the questions I ask may seem obvious, but this is because we want to give you the opportunity to tell us your experience in your own words. Some questions may also seem repetitive, but we want to make sure that we do not miss anything important. In the interest of time, I may need to stop you to move on to the next question. This is not because we are not interested in what you are saying; it is just because we do not want to run out of time.

Our conversation will be recorded so that we can accurately represent what you are saying during the discussion in our research report. We will now begin recording. OK? Once the recording starts, I will repeat this question for the recording and also go through the consent form which you have already received.

If the participant does not wish for the interview to be recorded, the interviewer should not proceed with the interview.

**START RECORDING**

Today is [date]. This is participant ID [Insert number here].

Do I have your permission to record this interview? [YES/NO]

I’m first going to cover a few points to make sure you understand what we will discuss and your rights as a research participant:

**[READ INFORMED CONSENT STATEMENTS]**

I would like to clarify a few points before we begin:

- I am not a medical doctor, so I am not qualified to give medical advice. If you have any questions about your son’s condition as a result of our conversation today, I advise you to follow up with your regular doctor.
- There are no right or wrong answers, we understand that everyone has different experiences and we are interested in what you have to say.
- Please speak loudly enough to be heard for the recording.
- If during our conversation you tell me about any side effects or adverse events your son has experienced while on ataluren (Translarna), I will need to report this to PTC Therapeutics International Ltd. In order to do this, I will need to ask for your name and contact details (e.g. email, phone number or address) so they can follow up with you. If this is the case, I will ask for the details at the end of the interview.
- Do you have any questions before we begin?

This interview is divided into several sections, in the first section I will ask you a few background questions. I will then ask about how Duchenne impacted you and your son in the months before he started taking ataluren (Translarna), before moving on to ask how ataluren (Translarna) has impacted both of your lives.

# SECTION 1: BACKGROUND

So to start off, I would like to ask you some background questions. You may have already answered some of these in the socio-demographic questionnaire, but I want to make sure I’m not missing anything.

1. How old is your son?
2. Can you tell me a bit about your son’s diagnosis with Duchenne?

Prompts

- - How old was he when he first showed symptoms
  - How old was he when he was he diagnosed

1. When did he start taking ataluren (Translarna)?
2. Is he taking ataluren (Translarna) now?
3. [If no longer taking ataluren (Translarna)] When did he stop taking ataluren (Translarna)?
4. Can you tell me who else is in your family?

Prompts

- - Partner
  - Other children

# SECTION 2: EXPERIENCE WITH Duchenne PRE-ATALUREN (TRANSLARNA)

I would now like to move on to the second part of the interview, to learn about you and your son’s experience with Duchenne.

## Impact on the individual with Duchenne

| **NOTE TO INTERVIEWER:**  **Throughout this section, remind the participant to think about their son’s life in the months before he started taking ataluren (Translarna). Make a note of their son’s age when he started taking ataluren (Translarna) and ask for clarification if they use general timeframes, such as ‘when he was younger’.** |
| --- |

1. Tell me about your son’s Duchenne in the months before he started taking ataluren (Translarna).
2. Walk me through a typical day for your son in the months before he started taking ataluren (Translarna).

Prompts:

- - What was he able to do?
  - What was he unable to do?
  - How did he feel?
  - What was a good day like?
  - What was a bad day like?

| **NOTE TO INTERVIEWER:**  **Only ask questions 9 to 16 and associated prompts if the topic has not already been addressed in questions 7 or 8. Also only use prompts that are appropriate for the age of their son and his ambulatory status. Note the symptoms mentioned in the field notes template so you can return to them in the following sections.** |
| --- |

1. Tell me about the symptoms your son experienced in the months before he started taking ataluren (Translarna).

Prompts:

- - Symptoms stable declining/improving

1. Tell me about his lower limb function in the months before he started taking ataluren (Translarna).

Prompts:

What about…

- - Getting up off the floor
  - Sitting
  - Standing
  - Walking
  - Running
  - Climbing stairs
  - Clumsiness/falling
  - Assisted/unassisted – how assisted?
  - [if unable] when did he lose the ability to sit/stand/walk/run/climb stairs?
  - Any pain when doing these?

1. Tell me about his upper limb function in the months before he started taking ataluren (Translarna).

Prompts:

What about…

- - Grasping objects?
  - Manipulating objects with his fingers?
  - Turning a tap on and off?
  - Doing up a button?
  - Lifting light/heavy objects?
  - Reaching for objects or slide an object across a table?
  - Lifting his arms above his head?
  - Transferring from one position to another using his hands?
  - Any pain when doing these?

1. Tell me about any fractures or broken bones he experienced in the months before he started taking ataluren (Translarna).

Prompts:

- - Lower body e.g. legs, pelvis
  - Upper body e.g. wrist, shoulder, skull
  - Pain
  - Healing

1. Tell me about his cognitive functioning (e.g. learning, communication, memory) in the months before he started taking ataluren (Translarna).

Prompts:

What about…

- Learning difficulties?
- Communication?
- Memory?
- Concentration?

1. Tell me about any behavioural issues in the months before he started taking ataluren (Translarna)?
2. Tell me about his sleep in the months before he started taking ataluren (Translarna).

Prompts:

- Sleep quality
- Daytime sleepiness
- Tiredness/fatigue
- Triggers

1. Tell me about any pain or discomfort he experienced in the months before he started taking Translara.

Prompts:

- Location
- Triggers
- Frequency
- Severity

1. Tell me about any other symptoms or issues he experienced in the months before he started taking ataluren (Translarna).

Prompts:

- Other muscle weakness e.g. in the face?
- Lung/breathing difficulties?
- Heart problems?
- Swallowing issues?
- Vision?
- Shivering/temperature regulation?
- Chest infections/colds?

1. Tell me about how Duchenne impacted your son’s life in the months before he started taking ataluren (Translarna).

| **NOTE TO INTERVIEWER:**  **Note to interviewer: Only ask questions 19 to 21 if the topic has not already been addressed in question 18 or elsewhere. Also only use prompts that are appropriate for the age of their son and his ambulatory status. Note the impacts mentioned in the field notes template so you can return to them in the following sections.** |
| --- |

1. Tell me about any ways Duchenne impacted your son’s ability to carry out his daily activities in the months before he started taking ataluren (Translarna).

Prompts:

- - School
  - Hobbies
  - Self-care

1. Tell me about any ways Duchenne impacted your son’s emotional wellbeing in the months before he started taking ataluren (Translarna).

Prompts:

- - Anxiety/worry
  - Depression/low mood
  - Confidence/self-esteem
  - Frustration
  - Positive emotions – was he happy?

1. Tell me about any ways Duchenne impacted your son’s social activities in the months before he started taking ataluren (Translarna).

Prompts:

- - Friends
  - Family - siblings
  - Examples of able/unable

1. Of everything we’ve discussed so far, or anything we may have missed, what would you say had the biggest impact on your son’s quality of life in the months before he started taking ataluren (Translarna)?

## Impact on the caregiver

| **NOTE TO INTERVIEWER:**  **Throughout this section, remind the participant to think about their life in the months before their son started taking ataluren (Translarna). Ask for clarification if they use general timeframes, such as ‘when he was younger’.** |
| --- |

1. During the same time period we have just been discussing, tell me about your experience of caring for your son.
2. Walk me through your typical day as a caregiver in the months before your son started taking ataluren (Translarna)?

Prompts:

- - How did you feel?
  - What was a good day like for you as a caregiver?
  - What was a bad day like for you as a caregiver?

| **NOTE TO INTERVIEWER:**  **Only ask questions 25 to 29 if the topic has not already been addressed in questions 23 or 24 or elsewhere. Also only use prompts that are appropriate for the age of their son and his ambulatory status. Note the impacts mentioned in the field notes template so you can return to them in the following sections.** |
| --- |

1. Tell me about how Duchenne impacted your life as a caregiver in the months before he started taking ataluren (Translarna).

Prompts:

- - Negative impacts
  - Positive impacts

1. Tell me about any ways Duchenne impacted your physical health in the months before he started taking ataluren (Translarna).

Prompts:

- - Physical symptoms; back pain, muscle soreness from carrying
  - Sleep, tiredness

1. Tell me about any ways Duchenne impacted your daily activities in the months before he started taking ataluren (Translarna).

Prompts:

- - Work; stopped working or working part-time
  - Hobbies
  - Self-care
  - House impact: home adaptations, moving house, car, bills
  - Cooking; steroid-friendly diet, swallowing issues

1. Tell me about any ways Duchenne impacted your emotional wellbeing in the months before he started taking ataluren (Translarna).

Prompts:

- - Anxiety/worry/stress
  - Depression/low mood/guilt
  - Confidence/self-esteem
  - Frustration
  - Positive emotions

1. Tell me about any ways Duchenne impacted your social activities in the months before he started taking ataluren (Translarna).

Prompts:

- - Friends
  - Family
  - Social activities
  - Relationship with partner

1. Tell me about how Duchenne impacted the rest of your family in the months before he started taking ataluren (Translarna).

Prompts:

- - Negative impacts
  - Positive impacts

1. Of everything we’ve discussed so far, or anything we may have missed, what would you say had the biggest impact on your quality of life in the months before he started taking ataluren (Translarna)?

# SECTION 3: IMPACT OF ATALUREN (TRANSLARNA)

I would now like to move on to the next part of the interview, to learn about you and your son’s experience with ataluren (Translarna).

## Impact on the individual with Duchenne

| **NOTE TO INTERVIEWER:**  **Throughout this section, remind the participant to think about their son’s life when taking ataluren (Translarna). Adapt the question depending on whether he is taking it now or was previously taking it (since/when).** |
| --- |

1. What led to your son taking ataluren (Translarna)?
2. What factors did you consider when making the decision to start treatment with ataluren (Translarna)?

Prompts:

- - Any changes to your son’s health?

1. What were your expectations of treatment with ataluren (Translarna) before your son started treatment?

| **NOTE TO INTERVIEWER:**  **Only ask question 35 to those who are still taking ataluren (Translarna).** |
| --- |

1. What are your expectations of treatment with ataluren (Translarna) now and looking ahead?
2. Thinking about all the symptoms and impacts we have discussed pre-ataluren (Translarna), tell me about your son’s experience with ataluren (Translarna).

Prompts:

- - Use field notes to prompt on symptoms/impacts mentioned in section 2.
  - Positive changes
  - Negative changes
  - No change
    - If no change, how was this perceived?

1. Walk me through a typical day for your son since he started taking/when your son was taking ataluren (Translarna).

Prompts:

- - What is/was he able to do?
  - What is/was he unable to do?
  - How does/did he feel?
  - What is/was a good day like?
  - What is/was a bad day like?
  - If no change, how did the day compare to pre-ataluren (Translarna)?

| **NOTE TO INTERVIEWER:**  **Only ask questions 38 to 42 if the topic has not already been addressed in questions 36 or 37 or elsewhere. Use your field notes to ensure that you prompt on all symptoms and impacts mentioned in section 2.** |
| --- |

1. Tell me about any ways in which ataluren (Translarna) (has) impacted your son’s symptoms.

Prompts:

- - Prompt on symptoms mentioned in section 2.

1. What is/was the overall impact of ataluren (Translarna) on your son?
2. Tell me about any ways in which ataluren (Translarna) (has) impacted your son’s daily activities?

Prompts:

- - Prompt on impacts mentioned in section 2.

1. Tell me about any ways in which ataluren (Translarna) (has) impacted your son’s emotional wellbeing?

Prompts:

- - Prompt on impacts mentioned in section 2.

1. Tell me about any ways in which ataluren (Translarna) (has) impacted your son’s social activities?

Prompts:

- - Prompt on impacts mentioned in section 2.

1. Tell me about any unexpected impacts of ataluren (Translarna) on your son’s life.

Prompts:

- - Positive
  - Negative

1. Tell me about any other impacts of ataluren (Translarna) on your son’s life that we have not discussed?

Prompts:

- - Positive
  - Negative

## Impact on the caregiver

| **NOTE TO INTERVIEWER:**  **Throughout this section, remind the participant to think about their life when their son is/was taking ataluren (Translarna). Adapt the question depending on whether he is taking it now or was previously taking it (since/when). Use your field notes to ensure that you prompt on all symptoms and impacts mentioned in section 2.** |
| --- |

1. Tell me about your experience of caring for your son since he started taking ataluren (Translarna)/when he was taking ataluren (Translarna).

Prompts:

- - Prompt on impacts mentioned in section 2.

1. Tell me about any unexpected impacts of ataluren (Translarna) on your life.

Prompts:

- - Positive
  - Negative

1. Tell me about any impacts of ataluren (Translarna) on the rest of your family.

Prompts:

- - Positive
  - Negative

1. Tell me about any other impacts of ataluren (Translarna) on your life that we have not discussed?

Prompts:

- - Positive
  - Negative

1. Would you recommend ataluren (Translarna) to another family with Duchenne?

Prompts:

- - Why/why not?

# SECTION 4: POST-ATALUREN (TRANSLARNA) (IF APPLICABLE)

| **NOTE TO INTERVIEWER:**  **Only ask these questions to those whose son has stopped taking ataluren (Translarna).** |
| --- |

## Impact on the individual with Duchenne

1. Why did your son stop taking ataluren (Translarna)?

Prompts:

- - Whose idea was it to stop treatment?
  - Did he experience any side effects/adverse events?
  - Did he become non-ambulatory
  - Were there any changes to his health?

1. Tell me about your son’s symptoms since he stopped taking ataluren (Translarna).

Prompts:

- - Prompt on symptoms mentioned in sections 1 and 2.

1. Tell me about any impacts on your son’s life since he stopped taking ataluren (Translarna).

Prompts:

- - Prompt on impacts mentioned in sections 1 and 2.

## Impact on the caregiver

1. Tell me about your experience of caring for your son since he stopped taking ataluren (Translarna).

Prompts:

- - Prompt on impacts mentioned in sections 1 and 2.

# INTERVIEW CLOSE

We are now coming to the end of this interview. Before we finish, do you have anything else you’d like to say about Duchenne or ataluren (Translarna)?

[If appropriate based on the interview] Would you be happy for your son to take part in a short interview about his experience with Duchenne and ataluren (Translarna)?

[If yes] Is he able to provide informed assent and talk to us for 10min-30mins via skype or able to attend face-to-face?

[If participant is suitable] Would you be happy for us to contact you about further interviews about your experience with Duchenne and ataluren (Translarna)?

We really appreciate the time you have taken to participate in this study.

Thank you, this concludes the interview.

**STOP RECORDING**

Confirm details of payment for interview.

[If applicable] Arrange follow-up interview with son.

[If applicable] Collect details of side effects or adverse events.
