# Supplementary material for: A qualitative study on the impact of caring for an ambulatory individual with nonsense mutation Duchenne muscular dystrophy
Source: J Patient Rep Outcomes. 2021 Aug 10;5:71. doi: 10.1186/s41687-021-00344-8 (PMC8353428; doi:10.1186/s41687-021-00344-8)
Supplement: Supplementary file 2 — Additional file 2: Supplementary file 2. Background questionnaire. [file 41687_2021_344_MOESM2_ESM.docx]

# Caregiver socio-demographic and clinical questionnaire

### About you

1. What is your sex? Please select one option.

- Male
- Female
- Other
- Prefer not to answer

1. Which of the following best describes your highest level of education? Please select one option.

- No formal qualifications
- ONC/BTEC
- O level/GSCE or equivalent
- A Level or Highers
- Higher education below degree level
- University degree or higher
- Other, please specify: ____________________
- Prefer not to answer

1. Which of the following best describe your ethnic background? Please select one option.

- White
- Mixed or multiple ethnicity
- Asian or Asian British
- Black, African, Caribbean or Black British
- Other, please specify: ____________________
- Prefer not to answer

1. How would you describe your employment status? Please select one option.

- Employed full-time
- Employed part-time
- Self employed
- Full-time homemaker/caregiver
- Retired
- Seeking work/unemployed
- Long term sick leave
- In education or training
- Other, please specify: ____________________
- Prefer not to answer

The following questions are about the care you provide for the individual with nonsense mutation Duchenne muscular dystrophy (nmDMD).

1. On average, how many hours a week do you spend providing practical and emotional care for them?

| Practical care | ___________ hours per week |
| --- | --- |
| Emotional care | ___________ hours per week |

1. Have you had to do any of the following in order to care for them? Please select all that apply.

- Stop working
- Reduce your working hours
- Change jobs
- None of the above

1. Does anyone else help care for this individual?

- Yes
- No

1. [If ‘yes’ to question 7] Who else helps care for them? Please select enter the number of hours per week that they spend providing practical and emotional care. If they do not provide any care, please enter ‘0’.

|  | **Practical care** | **Emotional care** |
| --- | --- | --- |
| Other parent/partner | ___________ hours per week | ___________ hours per week |
| Other family member | ___________ hours per week | ___________ hours per week |
| Friends | ___________ hours per week | ___________ hours per week |
| Paid personal assistant | ___________ hours per week | ___________ hours per week |
| Other, please specify:  ____________________ | ___________ hours per week | ___________ hours per week |

### Other than yourself and the individual you care for with nmDMD, who else is in your family?

- Partner
- Other children
- Other, please specify: ____________________

### About the individual you care for with nmDMD

The following questions are about the individual you care for with nmDMD.

1. How old is the individual you care for with nmDMD? ____________________
2. How old were they when you **first noticed their nmDMD symptoms**?

____________________years ____________________months

- Don’t know/can’t remember

1. How old were they when **formally diagnosed** with nmDMD?

____________________years ____________________months

- Don’t know/can’t remember

1. How did they get access to ataluren (Translarna™)?

- Managed Access Agreement (MAA)
- Compassionate use
- Don’t know/can’t remember

1. How old were they when they **first started** taking ataluren (Translarna™)?

____________________years ____________________months

- Don’t know/can’t remember

1. Are they **currently** taking ataluren (Translarna™)?

- Yes
- No

1. [If ‘No’ to question 15] How old were they when they **stopped** taking ataluren (Translarna™)?

____________________years ____________________months

- Don’t know/can’t remember

1. Are they **currently** taking steroids for their nmDMD?

- Yes
- No

1. [If ‘no’ to question 17] Did they take steroids for their nmDMD **before starting** ataluren (Translarna™)?

- Yes
- No

1. [If ‘no’ to question 15] Did they take steroids for their nmDMD **while taking** ataluren (Translarna™)?

- Yes
- No

1. Can they stand up from lying down (rise from supine)?

- Yes
- No

1. Can they stand unassisted?

- Yes
- No

1. [If ‘yes’ to question 21] Can they walk 10 metres?

- Yes, unassisted
- Yes, assisted
- No

1. [If ‘yes’ to question 22] Have they ever completed the six-minute walk test?

- Yes
- No
- Don’t know/can’t remember

1. [If ‘yes’ to question 23] What was the result and date of their most recent test?

- < 300 metres
- 300-400 metres
- > 400 metres

Approximate date of most recent test: ____________________

- Don’t know/can’t remember

1. [If ‘yes’ to question 22] Have they ever completed the North Star Ambulatory Assessment (NSAA)?

- Yes
- No
- Don’t know/can’t remember

1. [If ‘yes’ to question 25] What was the result and date of their most recent test?

Score of most recent test (range 0-34): ____________________

Approximate date of most recent test: ____________________

- Don’t know/can’t remember

1. [If ‘no’ to question 22] How old were they when they stopped being able to walk 10 metres?

____________________years ____________________months

1. Can they transfer themselves from one seated position to another? Please select the response which best describes the majority of transfers.

- Yes, unassisted
- Yes, assisted
- No

1. Do they have sideways curvature of the spine (scoliosis)?

- Yes
- No

1. Do they require ventilation?

- No
- Yes - at night-time
- Yes - during the day
- Yes – during the day and at night

1. Have they ever had their Forced Vital Capacity measured (this is the total amount of air exhaled in a Forced Expiratory Volume/FEV test)?

- Yes
- No

1. [If ‘yes’ to question ‘31’] What was the result and date of their most recent test?

- < 30
- 30 – 49
- 50+
- Don’t know/can’t remember

Approximate date of most recent test: ____________________

- Don’t know/can’t remember

1. Can they raise their hands to their mouth?

- Yes
- No
